# Impact of TiO_2_, ZnO, and Ag nanoparticles on anammox activity in enriched river Nile sediment cultures: unveiling differential effects and environmental implications

**DOI:** 10.1186/s12866-024-03603-y

**Published:** 2024-11-11

**Authors:** Mohamed A. Abd EL-Aziz, Ali M. Saeed, Mohamed K. Ibrahim, Wael S. El-Sayed

**Affiliations:** Microbiology Department, Faculty of Science, Ain Shams University, Cairo, Egypt

**Keywords:** Anammox bacteria, River Nile, Polluted sediments, 16S rRNA gene, *hzo* gene, Nanoparticles

## Abstract

**Background:**

The increasing use of nanoparticles (NPs) necessitates investigation of their impact on wastewater treatment processes, particularly anammox, a critical biological nitrogen removal pathway. This study explored the effects of short-term exposure to TiO_2_, ZnO, and Ag-NPs on anammox activity in enriched cultures derived from River Nile sediments.

**Materials and methods:**

Anammox bacteria were identified and enriched, with activity confirmed through 16S rRNA and hydrazine oxidoreductase (*hzo*) gene amplification and sequencing. Activity assays demonstrated efficient ammonium removal by the enriched culture. Subsequently, the impact of different sized and concentrated NPs on anammox activity was assessed.

**Results:**

XRD analysis confirmed NP behavior within the microcosms: TiO_2_ transformed, ZnO partially dissolved, and Ag remained ionic. *hzo* gene expression served as a biomarker for anammox bacterial activity. Interestingly, 100 nm TiO_2_-NPs up-regulated *hzo* expression, potentially indicating a non-inhibitory transformed phase. Conversely, ZnO and Ag-NPs across all sizes and concentrations significantly down-regulated *hzo* expression, suggesting detrimental effects. Ag-NPs amended microcosms showed a significant reduction (79%) in *hzo* gene expression and a detrimental effect on bacterial populations. Overall, anammox activity mirrored *hzo* expression patterns, with TiO_2_ (21 and 25 nm, respectively) exhibiting the least inhibition, followed by ZnO and Ag-NPs.

**Conclusion:**

This study highlights the differential effects of NPs on anammox, with the order of impact being Ag > ZnO > TiO_2_. These findings provide valuable insights into the potential environmental risks of NPs on anammox-mediated nitrogen cycling in freshwater ecosystems.

## Introduction

Microbial denitrification was historically considered the sole driver of N_2_ production in the environment [1]. However, the discovery of anammox, a process where anoxic ammonium oxidation with nitrite yields N_2_, revealed its significant contribution in marine ecosystems [2]. Anammox bacteria (Planctomycetes) utilize nitrite as an electron acceptor for ammonium oxidation, generating N_2_ via hydrazine (N_2_H_4_) as an intermediate [3]. Hydrazine dehydrogenase (HZO) is a key enzyme in this pathway, oxidizing N_2_H_4_ to N_2_ and potentially serving as a biomarker for anammox bacteria [4].

Anammox offers a promising alternative to conventional biological nitrogen removal (BNR) due to its high efficiency and low energy requirements [5]. This process is gaining traction in wastewater treatment plants (WWTPs) for increased biogas production and reduced aeration needs [5]. Anammox bacteria belong to the order Brocadiales within Planctomycetes, with 14 identified species [6]. Culture-independent methods like 16S rRNA analysis are crucial for their detection [7].

Anammox bacteria, previously thought to be confined to marine environments, are increasingly detected in freshwater habitats, including rivers, lakes, and oxygen minimum zones [2]. They can contribute significantly to nitrogen removal, with estimates suggesting up to 40% loss [8]. However, our understanding of freshwater anammox is limited, particularly regarding the influence of environmental factors like temperature, organic carbon, substrate availability, and salinity [9]. Elucidating these interactions is crucial for assessing the impact of human activities on freshwater quality.

Nanomaterials, with their novel properties, are widely employed for consumer and industrial products [10]. Nanoparticles (NPs), particularly metal oxides, have garnered significant research interest due to their diverse industrial and medical applications [11]. However, concerns regarding their potential detrimental effects on ecosystems and microbial processes are rising [12]. The unique size, quantum effects, and high surface area of NPs can lead to complex interactions with organisms, potentially causing unforeseen biological impacts [13]. Therefore, understanding NP interactions with microorganisms and their influence on biogeochemical cycles is crucial for environmental protection.

The EPA prioritizes monitoring NPs due to their potential environmental risks [14]. Their unique properties complicate toxicity assessment, especially in complex ecosystems [14]. Silver nanoparticles (Ag-NPs) are a concern, with studies showing inhibition of nitrification but limited data on freshwater anammox [15]. Similarly, knowledge gaps exist regarding the long-term effects of zinc oxide nanoparticles (ZnO-NPs) and the environmental behavior of titanium dioxide nanoparticles (TiO_2_-NPs) on anammox bacteria [16, 17]. Elucidating these impacts is critical for informed regulation of NPs. Shock-loading of TiO_2_-NPs at various concentrations (1-200 mg L^-1^) in AGS and AB-AGS bioreactors had varying effects on nutrient removal and microbial activity. Low concentrations (≤ 10 mg L^-1^) had no significant impact due to increased EPS production. However, higher concentrations (50–200 mg L^-1^) led to gradual deterioration of bioreactor performance, particularly in ammonia-nitrogen removal [18]. GO-NPs also negatively affected nutrient removal, with significant decreases in ammonia-nitrogen and COD removal rates at higher concentrations. Nitrite and nitrate reduction rates remained relatively stable. These findings highlight the potential negative impacts of nanoparticle exposure on wastewater treatment processes. Further research is needed to understand the underlying mechanisms and develop strategies to mitigate these effects [19].

Nanomaterials’ unique properties hold promise for environmental remediation, but their environmental impact remains unclear [20]. NPs are released throughout their lifecycle, ending up in wastewater, landfills, and sludge [21, 22]. These concerns highlight the need to understand how NPs affect both living organisms and non-living components of the environment [22]. Therefore, this study investigates the potential effects of NPs on anammox, a natural microbial process essential for nitrogen cycling, particularly in polluted sediment microcosms.

## Materials and methods

### Sediment samples collection

**Fig. 1:**
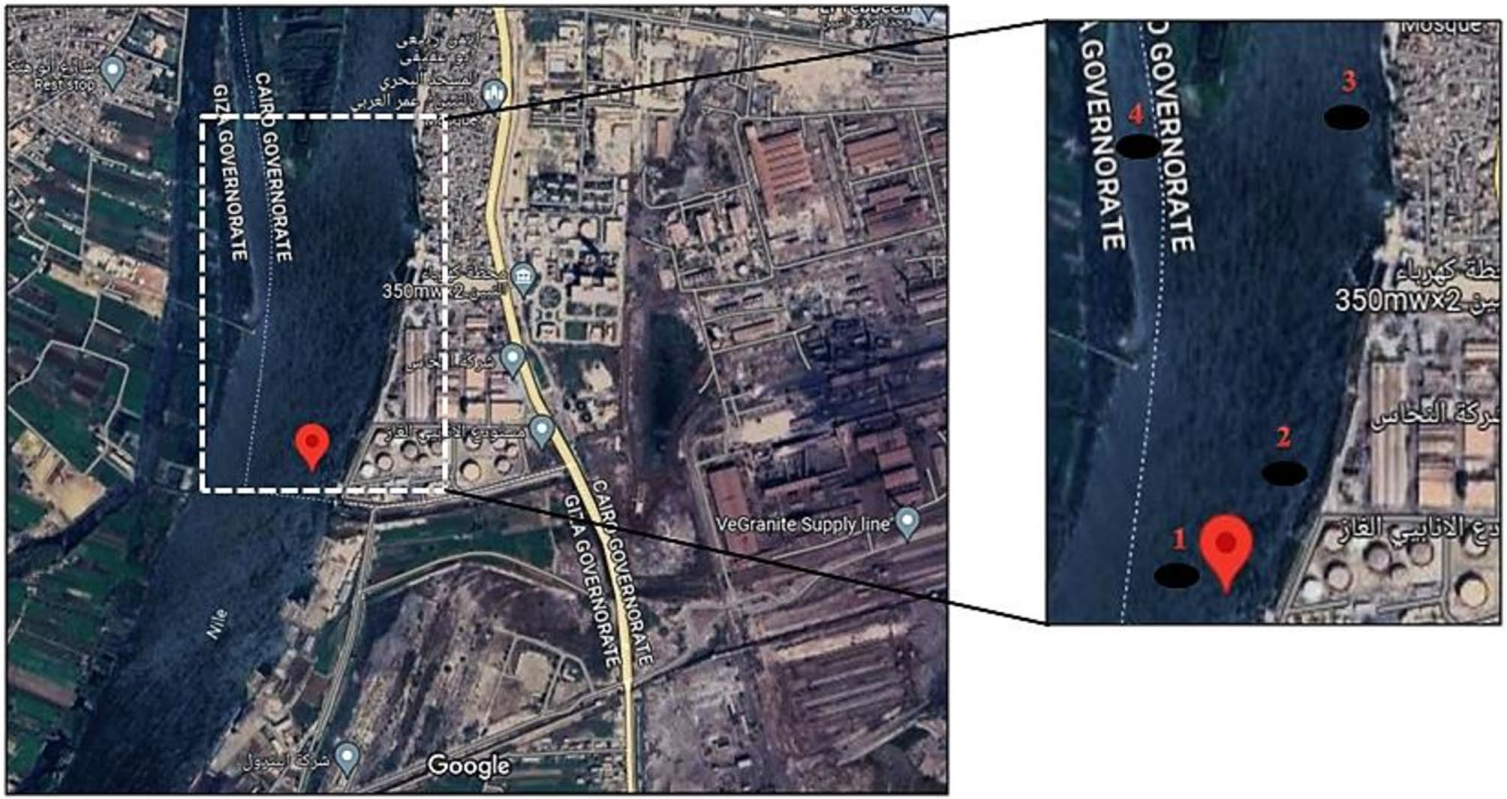
Geographical map showing the sampling sites at the River Nile at Tebin, Helwan industrialized area for the four samples addressed by this study

Polluted sediments were collected from four sites along the Nile River near Tebin, Helwan industrial area, Cairo Governorate (29° 46’ 16.1” N, 31° 17’ 25.7” E), known for high ammonium concentrations. Samples were collected in sterile, sealed plastic containers and transported on ice to the laboratory for anammox enrichment and microbiological analyses. A portion of each sample was stored at 4 °C in the dark until analysis. All samples were labeled according to collection site. Details of the sampling sites are provided in Fig. 1.

### Determination of physicochemical conditions of sediment

Sediment analysis included total nitrogen determination using a micro-Kjeldahl method with mixed acids [23]. Available phosphorus and ammonium were measured following standard methods (ASTM D3590-89B, ASTM D51588B) [24]. Total elements (Fe, Mn, Cu, Cd, Pb) were determined by aqua regia digestion and ICP-MS analysis [25, 26]. Available potassium (K) was quantified by flame photometry [25]. Sediment pH and electrical conductivity (EC) were measured using a pH meter and conductivity meter, respectively [25]. Total hydrocarbons were extracted with n-hexane and quantified by spectrophotometry at 430 nm [27]. Details on remaining analyses (e.g., As, Hg, Cr, Ni) can be found elsewhere [24, 26].

### Establishment of laboratory bioreactor

A stirred tank bioreactor (20 L) was established for anammox bacterial enrichment. A selective enrichment medium, formulated based on the anammox process [28], was used. This medium contained ammonium (NH_4_^+^) and nitrite (NO_2_^-^) as sole electron donor and acceptor, respectively, and bicarbonate (HCO_3_^-^) as a carbon source. The sterile reactor was initially filled with 5 L of the enrichment medium supplemented with NH_4_^+^ (0.396 g (NH_4_)_2_SO_4_) and NO_2_^-^ (0.414 g NaNO_2_). The synthetic basal medium [29] consisted of essential minerals and trace elements. River Nile polluted sediments (10 g L^-1^) and water were then inoculated. To maintain anaerobic conditions, the headspace was purged with CO_2_ and the reactor was sealed to prevent oxygen re-dissolution. The bioreactor was incubated at room temperature for one month before nucleic acid extraction.

### DNA extraction

Total genomic DNA was extracted from bioreactor sediment samples using a commercial soil DNA extraction kit (DNeasy Power Soil Kit/Soil Master DNA Extraction kit, QIAGEN, Hilden, Germany) following the manufacturer’s instructions. The extracted DNA was stored at -20 °C until further analysis. Polymerase chain reaction (PCR) amplification and subsequent 1% agarose gel electrophoresis with a 1.5 kb DNA ladder (Invitrogen, USA) were used to assess DNA quality and quantity. The gels were stained with ethidium bromide and visualized using a UV gel documentation system (Bio-Rad Laboratories Inc., CA, USA).

### PCR amplification

The 16S rRNA gene of anammox bacteria was amplified by polymerase chain reaction (PCR) using the primer set Brod541F (5′-GAGCACGTAGGTGGGTTTGT-3′) and Amx820R (5′-AAAACCCCTCTACTTAGTGCCC-3′) (M5) [30]. Two microliters of extracted DNA were used as template in a 50 µl reaction mixture containing 25 µl of pre-mixed PCR master mix (2X) (Genetix Biotech Asia Pvt. Ltd) according to the manufacturer’s protocol. Thermal cycling conditions consisted of an initial denaturation step at 95 °C for 5 min, followed by 33 cycles of denaturation (95 °C for 45 s), annealing (54 °C for 30 s), and extension (72 °C for 50 s). A final extension step at 72 °C for 10 min ensured complete amplicon formation [30].

The *hzo* gene was co-amplified with the previously described anammox 16S rRNA gene primers using specific primer sets hzocl1F1 (5′-TGYAAGACYTGYCAYTGG-3′) and hzocl1R2 (5′-ACTCCAGATRTGCTGACC-3′) (H1) [31], and hzoF1 (5′-TGTGCATGGTCAATTGAAAG-3′) and hzocl1R2 (5′-ACTCCAGATRTGCTGACC-3′) [32]. The 50 µl PCR reaction mixture contained template DNA (2 µl, 10 pg ^– 1^ µg), pre-mixed PCR master mix (25 µl), and primers (1 µl each). Thermal cycling involved initial denaturation (94 °C, 5 min), followed by 30 cycles of denaturation (95 °C, 1 min), annealing (50–53 °C, 1 min), and extension (72 °C, 2 min). A final extension step (72 °C, 10 min) ensured amplicon formation [31]. PCR products were visualized on 1% agarose gels stained with ethidium bromide after electrophoresis.

### Sequencing and phylogenetic analysis

Following PCR amplification, partial 16S rRNA and *hzo* gene sequences were obtained through Sanger sequencing. The 16S rRNA gene sequence was identified using the National Center for Biotechnology Information (NCBI) BLAST search program [33]. Sequence alignments were performed with CLUSTAL W 1.83 XP software, and a phylogenetic tree was constructed using the neighbor-joining method. The 16S rRNA gene sequence was then submitted to NCBI’s BankIt tool for accession number acquisition [34]. For the *hzo* gene, the obtained nucleic acid sequence was translated into amino acids, and the resulting protein sequences were aligned using CLUSTAL W 1.83 XP software. Phylogenetic analysis of the *hzo* gene was performed by constructing a tree using MEGA V6 software. Finally, the *hzo* gene sequence was submitted to NCBI’s BankIt tool for accession number acquisition [34].

### Determination of anammox activity

Anammox activity, defined as the nitrogen removal rate (sum of NH_4_^+^ and NO_2_^-^) under anoxic conditions with excess NH_4_^+^ and NO_2_^-^ [35], was assessed using the Nessler method. This colorimetric technique measures residual NH_4_^+^ concentration after anammox activity by forming a yellow-brown complex with NH_4_^+^ that absorbs light at 420 nm [36]. Daily sediment samples were collected from the bioreactor and a control (no sediment) over a 30-day incubation period to determine potential anammox rates. Nessler reagent was added to all samples, and the residual NH_4_^+^ concentration (indicating anammox activity) was measured spectrophotometrically at 420 nm using a UV-Vis spectrophotometer (Thermo Scientific, Waltham, MA, USA).

### Nanoparticles

Commercially sourced nanoparticles of zinc oxide (50,100 and 150 nm), silver (50,100 and 150 nm), and titanium dioxide (21, 25 and 100 nm) were anticipated to exhibit varying sizes and were procured from Sigma-Aldrich (Deisenhofen, Germany). Dynamic light scattering (DLS) employing a Zetasizer Nano ZS system (Malvern Instruments) was utilized to assess the size distribution of the nanoparticles. The surface charge, represented by zeta potential (ξ-potential), was determined via electrophoretic mobility measurements using the same instrument. The Smoluchowski approximation was then applied to convert the measured electrophoretic mobility into zeta potential values. Average values (intensity-weighted size and zeta potential) and standard deviations were reported based on data obtained from at least three independent samples. Total soluble metal concentration was quantified by graphite furnace atomic absorption (AA) spectrometry using a PerkinElmer 4100ZL GFAAS instrument. Prior to AA analysis, solid particles were eliminated from the suspensions using Amicon centrifugal ultrafiltration devices (Millipore Sigma) equipped with nominal 1–2 nm pore size cellulose membranes. Centrifugation was performed at 3500 rpm for 30 min in an Allegra X-15R centrifuge (Beckman Coulter Inc.) [14].

### Establishment of laboratory microcosms

Triplicate microcosms were established in 250 mL sterile Duran bottles. Each microcosm contained 90 g of sediment (anammox activity confirmed), 80 mL of selective enrichment medium (supplemented with ammonium and nitrite as previously described), and one of three nanoparticle (NP) types at varying concentrations (1, 5, and 10 µg mL^-1^). Continuous stirring ensured homogenous NP dispersion. After purging with CO_2_ to establish anaerobic conditions, all microcosms were incubated at room temperature for 3 months before RNA extraction. An unamended microcosm served as the control and followed the same incubation protocol.

### X-ray diffraction

Nanoparticles were characterized using x-ray diffraction technique (XRD). The XRD-patterns were used to certify the identity of each solid. The phases present were identified referring to JCPDS data, and particle size was estimated from Debye–Scherrer formula (Instrumental broadening), Eq. (1).

1 $$\:D=\frac{0.9{\lambda\:}}{\beta\:\:cos\:\theta\:\:}$$

where “λ” is wave length of X-Ray (0.1541 nm), “β” is FWHM (full width at half maximum, in radian), “θ” is the diffraction angle, and “D” is crystallite size [14].

The sediment slurries from the control and tested microcosms were examined by XRD at two stages, firstly, immediately after establishment of microcosms containing 1, 5, 10 µg mL^-1^ of Ag-NPs, ZnO-NPs, and TiO_2_-NPs (representing time zero; 16 − 1 series). Secondly, the sediments of all microcosms provided with same amounts of NPs at the end of incubation period of 3 months (16 − 4 series).

### Real-time quantitative PCR

Total RNA was isolated from sediment samples using the Invitrogen TRIzol^®^ RNA Isolation Reagent (Thermo Fisher Scientific) following the manufacturer’s instructions. RNA purity and concentration were assessed by measuring the A260/A280 nm and A260/A230 nm ratios using a NanoDrop 2000c spectrophotometer (Thermo Fisher Scientific). Isolated RNA was eluted in 20–50 µL nuclease-free water to a final concentration of 5 ng µL^-1^ and stored at -20 °C for subsequent analyses. cDNA synthesis was performed using the GeneDirex^®^ GScript First-Strand cDNA Synthesis Kit (Oligo dT) following the manufacturer’s protocol (GeneDirex, Inc, USA). The *hzo* gene expression levels, indicative of anammox activity, were quantified by RT-qPCR using BioEasy Master Mix (SYBR Green) kit (Hangzhou Bioer Technology Co., Ltd). Specific primer sets for *hzo* were hzoF1-hzocl1R2 [previously reported] and hzoF1 (5′-TGTGCATGGTCAATTGAAAG-3′) and hzoR1 (5′-CAACCTCTTCWGCAGGTGCATG-3′). The 16S rRNA gene served as an endogenous control with universal primers EUB341F (5′-TACGGGAGGCAGCAGCAG-3′) and EUB517R (5′-ATTACCGCGGC [14]. Each 25 µL reaction contained nuclease-free water, SYBR Green master mix (12.5 µL), primers (1.5 µL each, 20 µM), and cDNA (2 µL). The thermal cycling program consisted of: 50 °C for 1 min, 95 °C for 3 min, followed by 40 cycles of 95 °C for 15 s, 60 °C for 30 s, and 72 °C for 30 s. Relative quantification using the 2^(-ΔΔCT)^ method is a common approach for analyzing gene expression data from real-time quantitative PCR (qPCR) experiments. This method calculates fold change by comparing the target gene’s expression in treatment samples to a control group, normalized against a reference gene [37].

### Nucleotide sequence accession numbers

Sequences of anammox bacterial partial 16S rRNA gene amplified with primer set Brod541F/Amx820R from polluted River Nile sediments, have been deposited in GenBank under accession number PP448321 and the GenBank accession number for the *hzo* gene sequences amplified with primer set hzocl1F1/hzocl1R2 was MW010261.

### Statistical analysis

All statistical analysis in this study was carried out using analysis of variance (ANOVA, SPSS software version 18) followed by the Duncan test at 0.05 level. ^a-k^ means with different superscripts in the same column are considered statistically different (*P* ≤ 0.05). All data were calculated from at least 3 replicates and the standard error for each datum was plotted on the graph.

## Results

### Physicochemical conditions of the sediment

Analysis of the physicochemical properties of sediment samples revealed key factors potentially influencing anammox activity (Table 1). Notably, the sediments exhibited high concentrations of ammonium nitrogen (NH_4_^+^-N) at 400 mg kg^-1^ dry sediment, a crucial substrate for anammox bacteria. Additionally, the presence of total hydrocarbons (454.2 mg kg^-1^ dry sediment) suggests a potential source of organic carbon, which can indirectly support anammox activity by fueling the growth of heterotrophic bacteria that provide ammonium through organic matter degradation.

**Table 1:** Physicochemical conditions of the examined sediment samples

Environmental factors	Concentration (mg kg ^− 1^ dry sediment)
Total –N	0.18%
P	0.10%
K	0.12%
Mg	1.14%
Ca	3.28%
Fe	3.95%
NH_4_^+^-N	400
Mn	529.50
Zn	51
Cu	43.50
Co	15.70
Pb	105.50
Ni	118.20
As	13.50
Na	382
Cd	2.10
Se	< 1.3*
pH	9.56
EC	26 (dS m^− 1^)
Total hydrocarbon	454.2

*Detection limit (µg L^− 1^)

### Detection and phylogenetic analysis of anammox bacteria

**Fig. 2:**
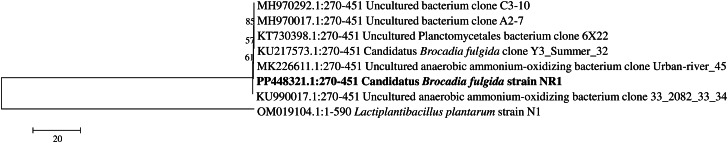
Phylogenetic tree of anammox bacteria based on partial 16S rRNA gene sequences in this study and representative database sequences

Genomic DNA extracted from sediment samples collected from the bioreactor was successfully amplified by PCR using the specific 16S rRNA gene primers Brod541F and Amx820R, targeting anammox bacteria [30]. Amplification yielded a product of approximately 280 base pairs (bp), consistent with the expected size for anammox 16S rRNA gene fragments. Phylogenetic analysis was performed on these positive amplicons alongside reference sequences for known anammox 16S rRNA genes. The maximum parsimony algorithm revealed high sequence identity (99%) between the obtained sequences and those of *Candidatus Brocadia* species (Fig. 2). This close phylogenetic relationship suggests the presence of anammox bacteria belonging to the *Candidatus Brocadia* genus within the sediment samples. The sequences were deposited in the National Center for Biotechnology Information (NCBI) under the accession name *Candidatus Brocadia fulgida* strain NR1.

### Detection and phylogenetic analysis of *hzo* gene

**Fig. 3:**
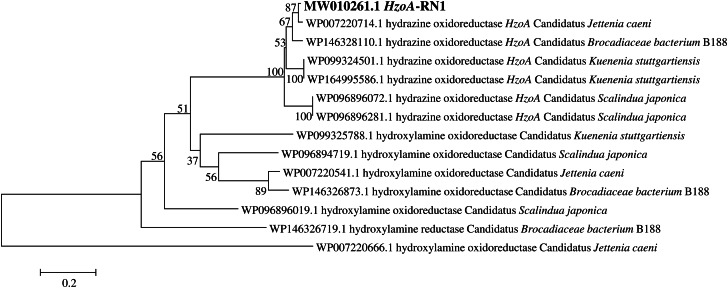
Phylogenetic tree constructed with distance and neighbour joining method from an alignment of *hzo* gene sequences with some reference phylotypes from GenBank

PCR amplification using primer sets hzocl1F1/hzocl1R2 and hzoF1/hzocl1R2 successfully targeted the *hzo* gene from the extracted DNA, yielding amplicons of approximately 470 bp and 600 bp, respectively [31, 32]. These fragment sizes align with the expected range for anammox *hzo* genes. Phylogenetic analysis was performed by constructing a tree using the amplified sequences alongside *hzo* gene sequences retrieved from GenBank (Fig. 3). The resulting tree positioned the experimental sequences within anammox *hzo* clusters, indicating their identity as functional *hzo* genes. Notably, these sequences exhibited high similarity (97%) to known *hzo* genes of *Candidatus jettenia* species. This close phylogenetic relationship suggests the presence of anammox bacteria harboring *hzo* genes closely related to *Candidatus jettenia* within the studied sediments. The obtained sequences were deposited in the National Center for Biotechnology Information (NCBI) under the accession name *Candidatus jettenia caeni* strain RN1.

### Assessment of anammox activity in bioreactor sediments

**Fig. 4:**
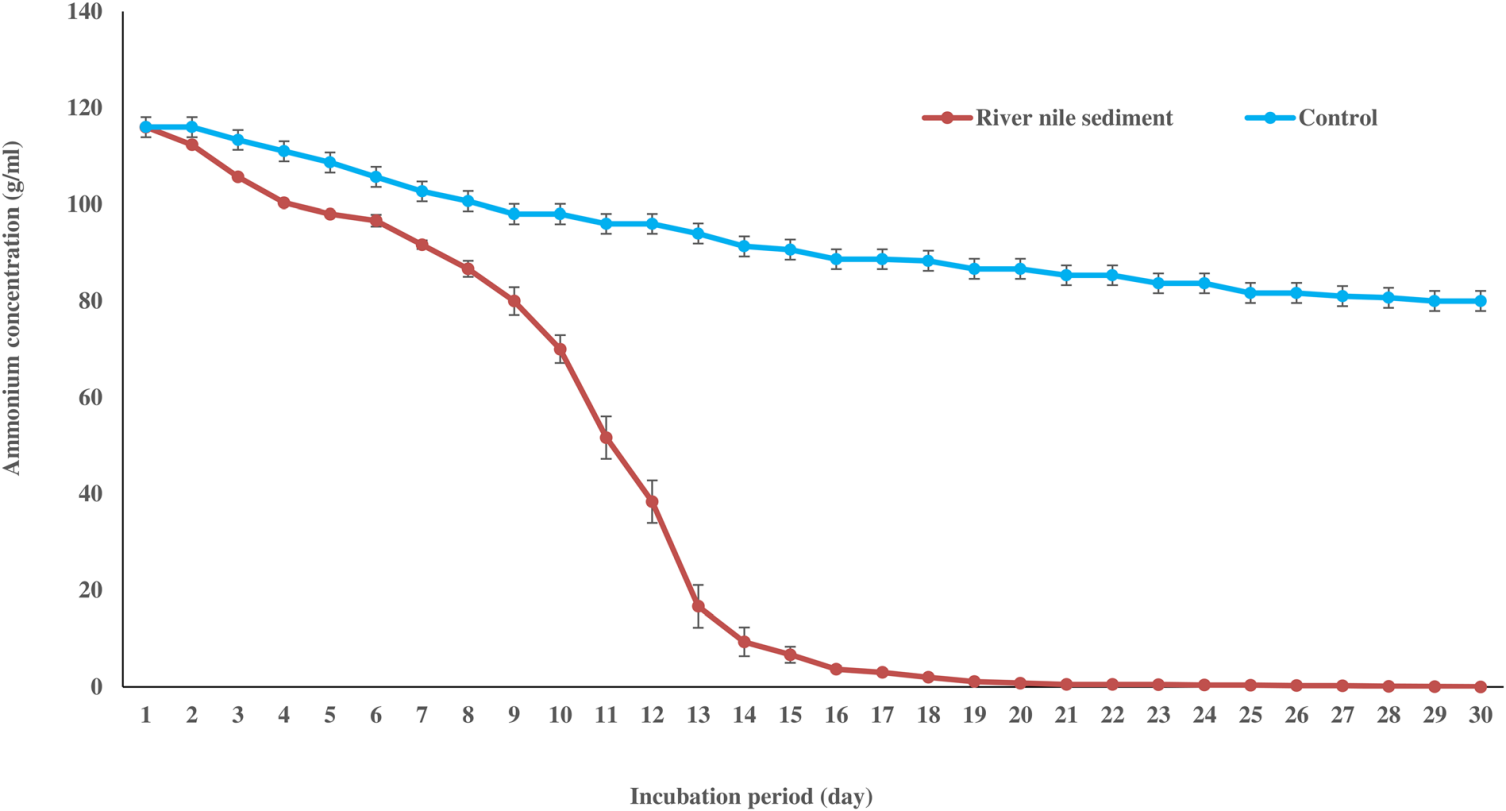
Difference in NH_4_^+^ concentration between control and sample with 30 days of incubation. Results represent mean ± SE of three triplicates. Control sample represents NH_4_^+^concentration in microcosm lacking sediment

The potential rates of anammox activity were assessed by monitoring the decrease in NH_4_^+^/NH_3_ concentration in sediment samples collected daily from the bioreactor over a 30-day incubation period. The initial NH_4_^+^/NH_3_ concentration was 68.22 mol g^-1^ dry sediment and it significantly decreased to 0.0587 mol g^-1^ dry sediment day⁻¹ by the end of the incubation. In contrast, the control samples (no sediment) exhibited minimal NH_4_^+^/NH_3_ removal, with concentrations ranging from 68.22 to 47.05 mol g^-1^ dry sediment per day. These observations suggest a high rate of anammox activity within the bioreactor sediments, characterized by a significant reduction in residual NH_4_^+^/NH_3_ over time compared to the control. As shown in Fig. 4, both the bioreactor and control samples initially displayed a steady-state NH_4_^+^/NH_3_ concentration during the early stages of the experiment. However, the bioreactor samples exhibited a gradual decrease in NH_4_^+^/NH_3_ concentration throughout the incubation period, whereas the control remained relatively stable. This differential pattern of NH_4_^+^/NH_3_ removal further supports the presence and activity of anammox bacteria within the bioreactor sediments.

### Anammox bacteria and functionally diverse microbial community in Nile river sediments

**Fig. 5:**
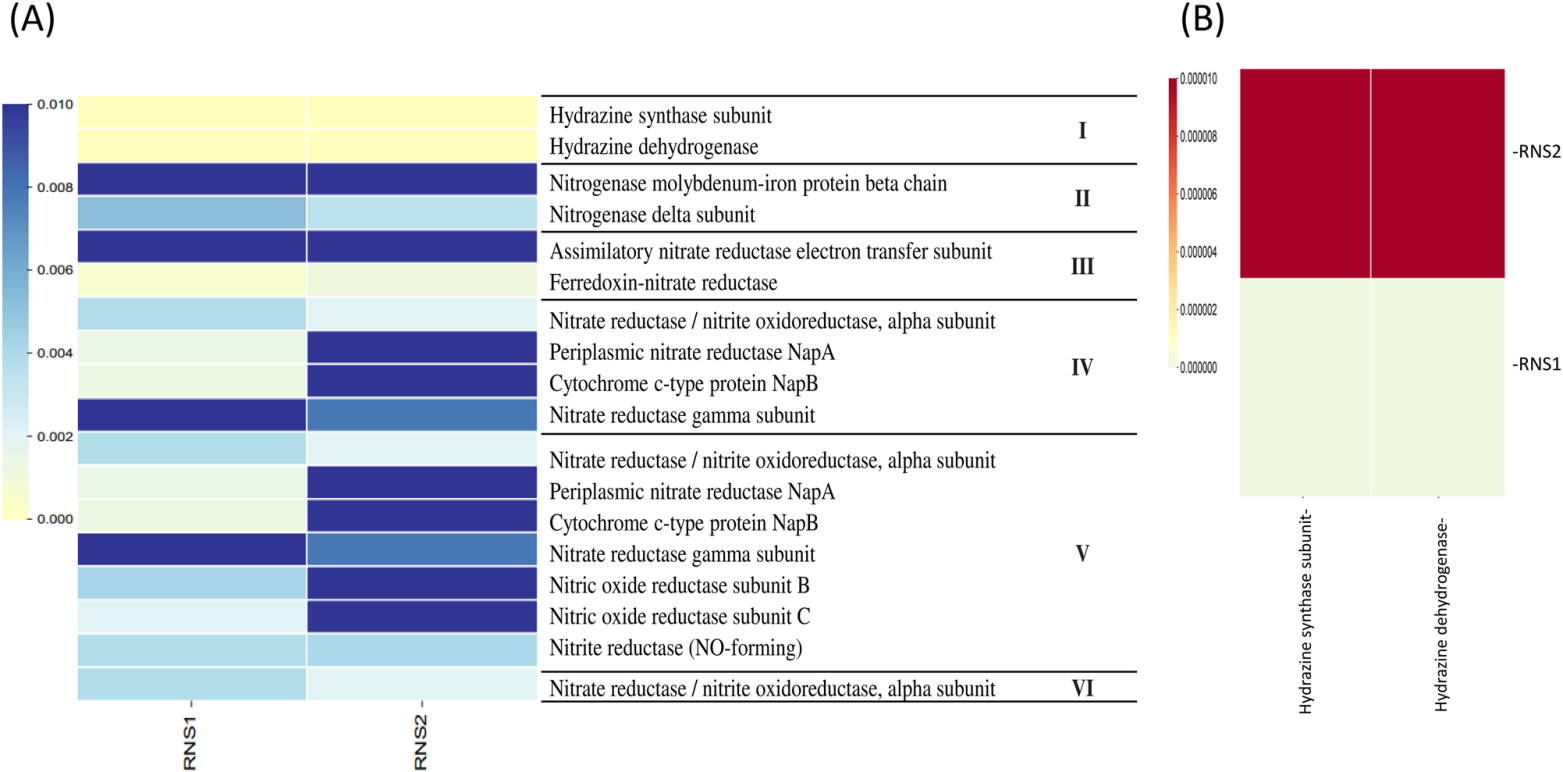
PICRUSt-constructed heatmap plot showing predictive functional biomarkers where (**A**) represents the major metabolic biomarkers identified as (I) Anammox, (II) Nitrogen fixation, (III) Assimilatory nitrate reduction, (IV) Dissimilatory nitrate reduction, (V) Denitrification, (VI) Nitrification, while (**B**) Heat map showing the two functional *hzo* genes identified by PICRUST tool this study. The heatmap was constructed using EZ-biocloud server based on taxonomic 16S rRNA gene sequences

PICRUSt functional prediction analysis, based on 16S rRNA gene sequencing data (Fig. 5A and B), identified genes encoding hydrazine synthase (KEGG KO K20933) and hydrazine dehydrogenase (KEGG KO K20935) as potential biomarkers for anammox bacteria within the sediment samples. These functional markers, along with the prior identification of anammox bacteria through taxonomic analysis and successful PCR amplification of the *hzo* gene, strongly suggest the presence and potential activity of anammox bacteria in the tested sediments. Additionally, PICRUSt revealed a diverse array of functional biomarkers indicative of various metabolic pathways within the sediment samples (Fig. 5A). Notably, genes associated with anaerobic processes, such as denitrification and dissimilatory nitrate reduction pathways, exhibited high relative abundances. These findings suggest a functionally diverse microbial community within the Nile River sediments, with a significant contribution of anaerobic metabolisms.

### Fate and bioavailability of nanoparticles in microcosms

**Fig. 6:**
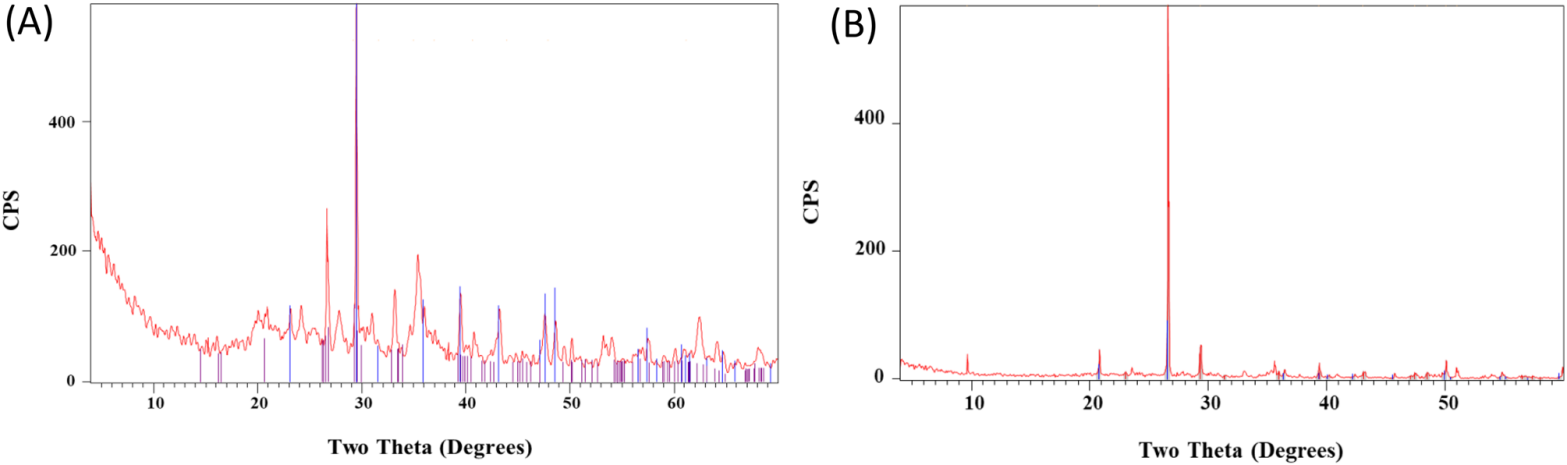
XRD pattern of microcosm sediment where (**A**) represents control sample lacking NPs at zero time, while (**B**) represents sediment microcosm supplemented with 10 µg mL^− 1^ Ag-NPs of size 150 nm at the end of the treatment period after 3 months of incubation

X-ray diffraction (XRD) analysis of the sediment revealed a predominantly amorphous phase, resulting in a low-intensity pattern at the usual scale. To extract meaningful information, the intensity scale was expanded to visualize distinct peaks. While the complex profile with variable peak characteristics posed challenges in definitive phase identification (Fig. 6A), some peaks could be assigned to specific crystalline phases using JCPDS files.

The most intense peak (100%) in the starting sediment (sample 16 − 1) at 2θ = 29.46° corresponds to the calcite phase of calcium carbonate (CaCO_3_). This rhombohedral calcite has lattice parameters of a = 4.98870 Å and b = 17.05290 Å (JCPDS file 83–0578). The second most intense peak (28.73%) at 2θ = 26.7° indicates the presence of the quartz phase of silicon dioxide (SiO_2_) (JCPDS file 46-1045). Tridymite, another polymorph of SiO_2_, is suggested by two peaks at 2θ = 33.17° and 35.45° (JCPDS file 18-1170).

In the sediment sample collected after the test period (sample 16 − 4), the relative abundance of quartz appears to have increased based on the presence of an additional peak at 2θ = 59.98° (33.95%). Furthermore, a 50% increase in quartz crystallite size was estimated by comparing the full width at half maximum (FWHM) of the peak at 2θ = 26.7°. The presence of tridymite is also inferred from the appearance of two peaks at 2θ = 20.94° and 23.27° (JCPDS file 18-1170). Notably, the calcite phase of CaCO_3_ remains the most abundant crystalline component (2θ = 29.48°, 100%), but with a noticeable decrease in crystallite size (Tables 2 and 3).

**Table 2:** Sediment (at zero time): [bold: quartz], [italic: tridymite], [bolditalic: Calcite]

Pos. [°2θ]	d-spacing [Å]	Height [cts]	FWHM Left [°2θ]	Rel. Int. [%]
**26.7026**	**3.33853**	**15.00**	**0.2362**	**28.73**
***29.4688***	***3.03114***	***52.23***	***0.0984***	***100.00***
*33.1703*	*2.70088*	*9.62*	*0.2558*	*18.42*
*35.4522*	*2.53209*	*13.33*	*0.6298*	*25.52*

**Table 3:** Sediment (after 3 months): [bold: quartz], [italic: tridymite], [bolditalic: Calcite]

Pos. [°2θ]	d-spacing [Å]	Height [cts]	FWHM Left [°2θ]	Rel. Int. [%]
*20.9397*	*4.24249*	*28.95*	*0.1181*	*50.38*
*23.2757*	*3.82173*	*28.78*	*0.1181*	*50.09*
**26.7173**	**3.33673**	**33.56**	**0.1181**	**58.41**
***29.4887***	***3.02915***	***57.46***	***0.1181***	***100.00***
*33.1551*	*2.70208*	*6.33*	*0.4723*	*11.02*
*35.4589*	*2.53163*	*11.47*	*0.4330*	*19.96*
**59.9852**	**1.54222**	**19.50**	**0.1181**	**33.95**

In all the patterns of all types of nanoparticles–treated samples no peaks due to added nanoparticles were detected, therefore, the analysis of the patterns concentrated on inspecting the changes in the peaks due to phases present in the sediment.

The impact of ZnO-NPs size and concentration on the mineralogy of the sediment was evaluated through XRD analysis. Compared to the starting sediment, the addition of ZnO-NPs (50 nm) primarily affected the silicon dioxide phases. Notably, the tridymite phase (peaks at 2θ = 20.94° and 23.27°) disappeared, and quartz abundance decreased (lack of peak at 2θ = 59.98°). Interestingly, the relative intensity of the dominant quartz peak exhibited a non-monotonic response with increasing ZnO content, suggesting complex interactions. However, a general trend of decreasing quartz crystallite size was observed with increasing ZnO content. Regardless of size, calcite remained the dominant crystalline phase at all ZnO concentrations.

For the 100 nm ZnO-NPs, no tridymite peaks were detected. The quartz crystallite size generally increased with increasing ZnO content (1–10 µg), while calcite crystallite size showed a tendency to increase until 5 µg of ZnO, with no further change observed.

At 150 nm, the calcite phase appeared most affected by ZnO at lower concentrations (up to 5 µg). Here, the major calcite peak displayed the lowest relative intensity compared to silica phases (likely cristobalite based on JCPDS file 39-1425, with 100% intensity). However, at 10 µg of ZnO, calcite abundance appeared higher than silica.

XRD analysis revealed contrasting effects of TiO_2_-NPs size and concentration on sediment mineralogy. For 21 nm nanoparticles, tridymite peaks were absent at both 1 and 5 µg TiO_2_ concentrations. Interestingly, at 1 µg, calcite appeared more affected than the silica phase. However, with increasing TiO_2_ content, calcite became dominant with a constant crystallite size. Notably, the remaining quartz exhibited a decrease in crystallite size with increasing titania content.

In contrast, 25 nm TiO2-NPs displayed no clear influence of increasing content on the various phases. Similarly, 100 nm nanoparticles lacked a systematic effect within the range of 1–5 µg. However, at 10 µg, both calcite and silica phases were significantly affected, as evidenced by the absence of 100% intensity peaks for either component.

For 50 nm Ag-NPs, increasing the concentration from 1 µg to 5 µg resulted in a decrease of dominance of calcite more than that of quartz, as indicated by changes in relative peak intensities. At 10 µg, calcite became the most affected phase, evidenced by a 100% relative intensity peak for quartz. Interestingly, quartz persistence was accompanied by an increase in crystallite size with increasing Ag content. (Table 4).

**Table 4:** Sediment amended with Ag (150 nm) (1 µg mL⁻¹)

Pos. [°2θ]	d-spacing [Å]	Height [cts]	FWHM Left [°2θ]	Rel. Int. [%]
20.8009	4.27049	18.75	0.2362	11.63
26.6023	3.35090	161.23	0.1181	100.00
29.4045	3.03762	72.20	0.0984	44.78
33.1178	2.70503	10.53	0.2755	6.53
35.4016	2.53559	9.70	0.4723	6.02
39.4379	2.28489	11.58	0.2755	7.18

In contrast, 100 nm Ag-NPs displayed a greater initial dominance of silica phases (tridymite and quartz) compared to calcite at 1 µg. As the concentration increased to 5 µg, additional quartz peaks emerged, suggesting a preference for quartz interaction. However, at 10 µg, tridymite disappeared, and both quartz and calcite displayed similar, near-100% relative peak intensities. Notably, no significant changes in crystallite size were observed for either silica or calcite phases across all Ag concentrations for 100 nm particles. (Table 5).

**Table 5:** Sediment amended with Ag (150 nm) (5 µg mL⁻¹)

Pos. [°2θ]	d-spacing [Å]	Height [cts]	FWHM Left [°2θ]	Rel. Int. [%]
20.8448	4.26159	10.01	0.4723	13.04
26.5344	3.35931	76.76	0.0787	100.00
29.3712	3.04100	47.70	0.1968	62.14
30.8349	2.89989	10.51	0.2362	13.69
33.0514	2.71032	10.04	0.2362	13.08
35.3486	2.53927	13.47	0.6298	17.55
39.3724	2.28854	9.81	0.3149	12.78

The impact of 150 nm Ag-NPs differed significantly (Table 6). Here, quartz displayed a dominant relative abundance compared to calcite throughout the experiment, suggesting a preferential interaction. This dominance coincided with an increasing trend in quartz crystallite size, while calcite crystallite size decreased with increasing Ag content. Interestingly, the overall crystallinity of the sediment appeared to improve at this size, as reflected by the increased heights of prominent peaks (Fig. 6B).

**Table 6:** Sediment amended with Ag (150 nm) (10 µg mL⁻¹)

Pos. [°2θ]	d-spacing [Å]	Height [cts]	FWHM Left [°2θ]	Rel. Int. [%]
9.6567	9.15162	24.24	0.0400	4.64
20.7816	4.27087	22.16	0.1068	4.24
26.5691	3.35223	522.61	0.0559	100.00
29.3449	3.04114	41.04	0.1286	7.85
39.3533	2.28771	10.15	0.1743	1.94

The limited crystallinity of the sediment presented a challenge for in-depth XRD analysis. However, some key observations were possible. Among the three nanoparticle types investigated (TiO_2_, Ag, ZnO), TiO_2_ exhibited the least impact on the sediment mineralogy. Conversely, Ag-NPs displayed the most pronounced influence, with variations in particle size affecting the extent of modification. ZnO-NPs demonstrated a moderate level of reactivity with the sediment, without a clear distinction in their effect based on size.

### Differential Regulation of *hzo* gene by diverse nanomaterials in anammox microcosms

**Fig. 7:**
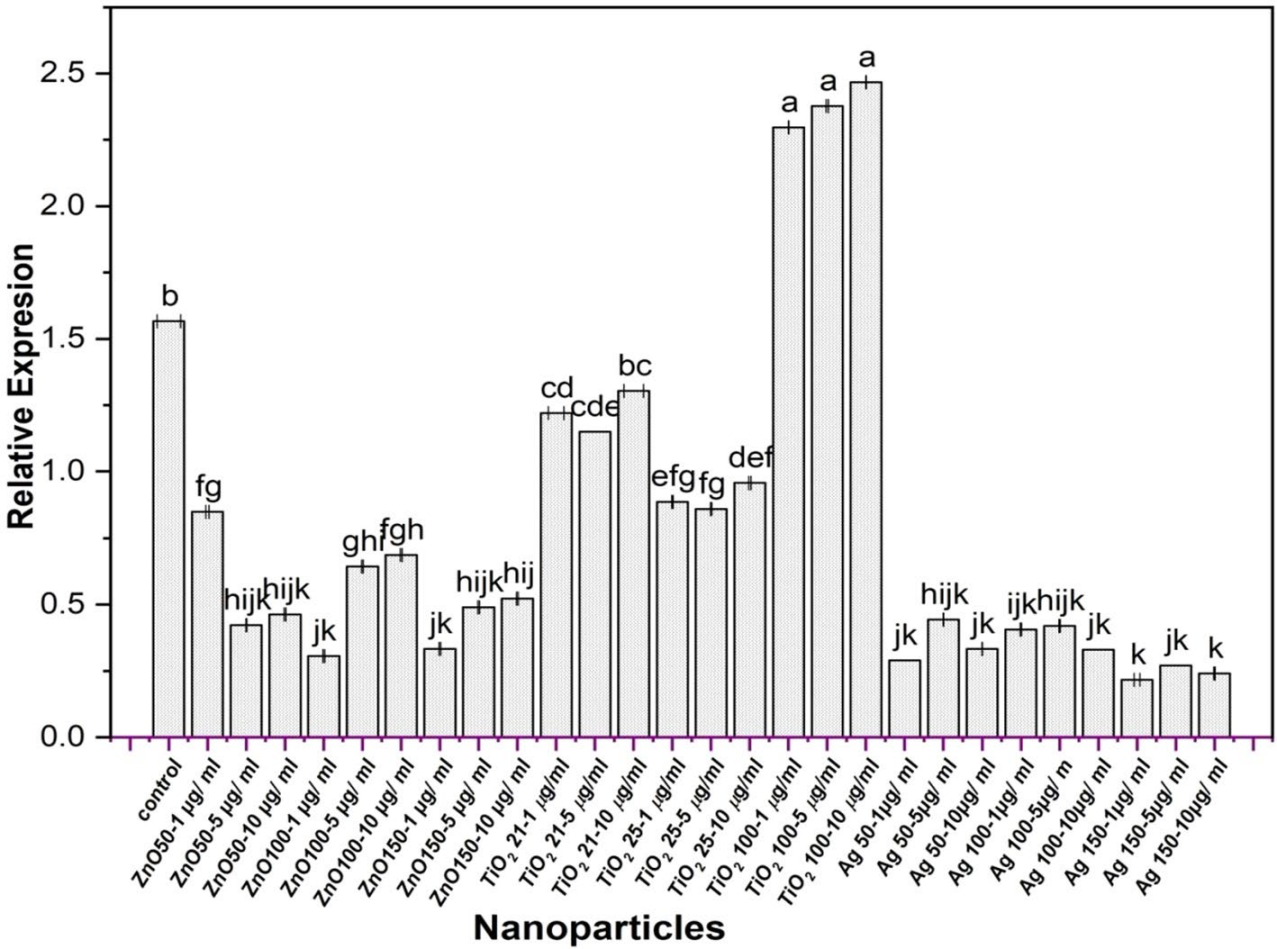
Comparative expression level of *hzo* in microcosms amended with different concentrations of TiO_2_-NPs, ZnO-NPs and Ag-NPs. 16S rRNA genes were used as an endogenous control. Control sample represents expression level in microcosm lacking NPs. Data are shown as the mean ± SE of triplicate measurements from independent experiments. Statistical significance was assessed using a one-way ANOVA (analysis of variance, SPSS software v.21) test and the means were compared with Duncan’s test at 0.05 level. Accordingly, mean values with different small letters (a-k) are considered statistically different (*P* ≤ 0.05)

Extracted total RNA from established microcosms exhibited acceptable quality, with final concentrations ranging from 25 to 264 ng µL^-1^. RT-qPCR analysis successfully amplified and quantified the target gene, *hzo*, along with the endogenous control gene, 16S rRNA. Melting curve analysis confirmed the absence of non-specific amplification in all reactions. The experiment aimed to assess the impact of various nanoparticle (NP) treatments on *hzo* gene expression by indigenous anammox bacteria within polluted Nile River sediment using RT-qPCR. As depicted in Fig. 7, the *hzo* gene expression profile varied significantly across sediment samples (after 3 months of incubation) depending on the type, size, and concentration of the applied NPs. Notably, *hzo* gene expression levels displayed significant differences between sediment samples amended with different sizes (21, 25, and 100 nm) of TiO_2_-NPs, (50, 100, and 150 nm) of ZnO-NPs, and Ag-NPs at concentrations of 1, 5, and 10 µg mL^-1^ compared to the unamended control. Treatment with ZnO-NPs (all sizes) at all concentrations (1, 5, and 10 µg mL^-1^) resulted in a substantial down-regulation (67%) of *hzo* gene expression and transcript abundance compared to the control. Exposure to 50 nm ZnO-NPs elicited a concentration-dependent modulation of *hzo* gene expression. While a concentration of 1 µg mL^-1^ exerted minimal inhibitory effects, a significant reduction in *hzo* transcript levels was observed at 5 and 10 µg mL^-1^. Microcosms treated with larger ZnO-NP sizes (100 and 150 nm) also displayed decreased *hzo* expression, with varying degrees of inhibition across different concentrations. Similarly, all Ag-NP treatments (all sizes and concentrations) caused significant down-regulation of gene expression compared to the control, with the lowest expression levels observed across all Ag-NP treatments (at least 79% down-regulation). Ag-NP treatment consistently inhibited *hzo* gene expression across all tested concentrations. However, the magnitude of this inhibitory effect was influenced by Ag-NP size. Particles of 150 nm diameter induced the most pronounced reduction in *hzo* transcript levels, followed by 100 and 50 nm particles. Interestingly, TiO_2_-NPs displayed a size-dependent effect. Treatment with 100 nm TiO_2_-NPs at all concentrations (1, 5, and 10 µg mL^-1^) led to a significant up-regulation (52%) of *hzo* gene expression compared to the control, whereas treatment with 21 and 25 nm TiO_2_-NPs resulted in down-regulation (22% and 42%, respectively) at all concentrations (1, 5, and 10 µg mL^-1^). In conclusion, the impact of nanoparticles on anammox activity and bacterial community structure depended on the type and concentration of the NPs, with the inhibitory effect following the order Ag > ZnO > TiO_2_.

### Impact of short-term exposure of nanoparticles on the anammox activity

Initially, all microcosms exhibited high *hzo* gene expression levels, indicating robust anammox activity. However, during the 3-month incubation period, microcosms amended with the three nanoparticle categories displayed a reduction in *hzo* gene expression compared to the control (unamended) and initial levels (zero time) within the amended microcosms. This suggests a potential inhibitory effect of the nanoparticles on anammox bacterial activity. The observed decrease in *hzo* gene expression likely translates to a decline in anammox activity within the nanoparticle-amended microcosms. Further studies are necessary to elucidate the specific mechanisms by which these nanoparticles might be inhibiting anammox bacteria.

## Discussion

Anammox, a microbially-mediated process within the nitrogen cycle, plays a crucial role in removing excess nitrogen from aquatic environments. This process contributes significantly to the elimination of unwanted environmental contaminants, ultimately leading to the production of inert dinitrogen gas (N_2_). The present study focuses on the potential of anammox activity in polluted sediments from the River Nile, collected at Tebin, Helwan industrialized area, Cairo Governorate, Egypt. This area receives industrial effluent, potentially leading to elevated ammonium concentrations - a key substrate for anammox bacteria. This selection aligns with previous studies by Wang et al. [38], who investigated anammox in sediments from the contaminated Fuhe River near Baoding, China, another environment likely impacted by anthropogenic activities.

The presence of anammox bacteria in the polluted Nile River sediments was confirmed through the amplification and sequencing of target genes. For 16S rRNA gene analysis, the primer set Brod541F-Amx820R (M5) yielded amplicons exhibiting 99% sequence identity to known anammox bacteria. This finding aligns with Li et al. [31], who reported high specificity (98%) for anammox detection using the same primer set. The closest phylogenetic affiliation of the obtained sequences was with *Candidatus Brocadia*, consistent with Zhang et al. [39] who observed dominance of this genus in freshwater environments. Similarly, the *hzo* functional gene analysis using primer set hzocl1F1-hzocl1R2 (H1) revealed 97% amino acid sequence similarity to reference sequences. This primer set al.so demonstrated efficiency and specificity for targeting *hzo* genes in sediment samples, as reported by Li et al. [31]. The obtained *hzo* sequences exhibited close relationships to *Candidatus jettenia*, in agreement with Wang et al. [38]. Notably, the co-existence of *Candidatus Brocadia* and *Candidatus jettenia* within the sediments suggests a diverse anammox bacterial community. This finding aligns with observations by Shen et al. [9] in a freshwater reservoir, Zhu et al. [8] in the Three Gorges Reservoir, and Sun et al. [40] in freshwater river and lake sediments.

Anammox activity was assessed by monitoring the residual NH_4_^+^/NH_3_ concentration in the samples using the Nessler reagent. A significant decrease (99%) in NH_4_^+^/NH_3_ concentration was observed over 30 days of treatment compared to the stable control samples. This depletion indicates a high rate of anammox activity, driven by the consumption of NH_4_^+^/NH_3_ over time. While the exact rates in this study cannot be directly compared to those reported by [41] (isotope tracer experiments in the Koisegawa River, Japan) due to methodological differences, both studies demonstrate the presence of anammox activity in freshwater sediments.

The impact of nanoparticles (NPs) on wastewater treatment processes, particularly those involving sensitive microbial communities, remains largely unexplored. While studies have established the ability of metal NPs to alter soil microbial diversity [42], the potential risks they pose to anammox bacteria, a crucial component of anaerobic ammonium oxidation (anammox) in wastewater treatment, are unknown. This knowledge gap necessitates the development of new approaches for environmental risk assessment of NPs [43]. Existing literature suggests that NPs can exert antimicrobial effects on specific soil microorganisms [44] or disrupt the overall microbial community structure [45]. In this context, we investigate the potential negative consequences of NPs on anammox activity within polluted sediments. Our study utilizes reverse transcription quantitative PCR (RT-qPCR) as a sensitive technique to assess the transcriptional regulation of the *hzo* gene, a well-established functional biomarker for anammox activity [46]. Hydrazine oxidoreductase (HZO), encoded by the *hzo* gene, is a key enzyme in the anammox pathway, responsible for dehydrogenating hydrazine to N_2_ gas [46]. Klotz et al. [47] proposed *hzo* as a functional phylogenetic marker due to its apparent universality among anammox bacteria [31]. This rationale justifies targeting the *hzo* gene via RT-qPCR to assess the impact of NPs on anammox bacterial activity in polluted sediments.

Two specific sets of primer pairs (hzoF1 and hzocl1R2) and (hzoF1 and hzoR1) were tested in this study to evaluate the expression level of *hzo* gene as an indicator for anammox activity potential in polluted sediments amended with NPs using RT-qPCR.

RT-qPCR analysis of *hzo* gene expression in sediments amended with 100 nm TiO_2_-NPs revealed a significant up-regulation (52%) after 3 months compared to the unamended control at all tested concentrations (1, 5, and 10 µg mL^-1^). Notably, this up-regulation was observed even at the highest concentration (10 µg mL^-1^) at the initial time point (zero time). Conversely, TiO_2_-NPs with sizes of 21 and 25 nm caused down-regulation of *hzo* gene expression in sediments after 3 months compared to the control at zero time. These findings partially align with Zhang et al. [17], who reported that low-concentration TiO_2_-NPs benefit anammox and enhance nitrogen removal, while high concentrations (50 mg L^-1^) have no impact due to NP aggregation. However, our results diverge from those of Z.Z. Zhang et al. [48] who observed no significant inhibition on specific anammox activity. Additionally, there is some disagreement with El-Sayed et al. [14], who showed no significant difference in gene expression in soil samples amended with high concentrations (10 µg mL^-1^) of TiO_2_-NPs compared to the control. The continued presence of anammox bacteria, *Candidatus Brocadia* and *Candidatus Jettenia*, in TiO_2_-NP treated sediments suggests that anammox activity in polluted sediments may exhibit resilience. Despite the known antimicrobial activity of TiO_2_-NPs [14], their reduced toxicity towards indigenous sediment microorganisms could be attributed to: (i) the inherent stability of rutile TiO_2_ compared to the anatase form [49], and (ii) the potential for interaction with other soil components, particularly heavy metals, which could limit their bioavailability [42]. As reported by Shah et al. [50], the observed variations in the effect of TiO_2_-NPs might be due to their transformation over time between rutile and anatase forms, leading to decreased interaction with heavy metals and increased bioavailability. This phenomenon could explain the diverse impacts of TiO_2_-NPs on bacterial species diversity in soil. Most studies have shown negative effects of ZnO-NPs and Ag-NPs on anammox bacteria and activity, while TiO2-NPs have exhibited less inhibitory effects. This may be due to TiO_2_-NPs tendency to form aggregates rather than dissolve into ions, potentially reducing their toxicity due to increased particle size [51]. Extracellular polymeric substances (EPS) produced by anammox bacteria can play a protective role against nanoparticles. While it was previously thought that TiO_2_-NPs might not accumulate on bacterial cell walls or EPS due to electrostatic repulsion [52], SEM analysis has shown their aggregation on anammox bacteria [48]. EPS can protect microorganisms from environmental stressors, including nanoparticles, and also plays a role in granulation and stability of anammox granules [53]. Despite the potential protective role of EPS, long-term exposure to TiO_2_-NPs can still negatively impact anammox activity. A study by T. Sari et al. [53] found that increasing TiO_2_-NP concentrations led to a gradual deterioration of anammox activity, with a significant inhibition observed at 300 mg L^-1^.

ZnO is a versatile inorganic compound exhibiting various crystalline structures [14]. Due to its high production volume, ZnO-NPs pose a significant environmental release risk [54]. Studies have shown that ZnO-NPs can exert selective toxicity towards certain beneficial soil microorganisms [45, 55]. In this study, ZnO-NPs of varying sizes (50, 100, and 150 nm) at both low (1 and 5 µg mL^-1^) and high (10 µg mL^-1^) concentrations significantly down-regulated *hzo* gene expression in sediment samples after 3 months compared to the unamended control at zero time. These findings suggest that ZnO-NPs, irrespective of concentration, can successively repress *hzo* gene expression within established microcosms. Our observations align with those of Zhang et al. [17], who reported concentration-dependent inhibition of anammox by ZnO-NPs. Notably, low concentrations (1 mg L^-1^) significantly suppressed anammox activity and nitrogen removal in their study. However, our results diverge from those of Z.Z. Zhang et al. [56] who observed no impact on anammox activity with ZnO-NPs up to 50 mg g^-1^ suspended solids (SS). There is also partial agreement with Frenk et al. [57], who demonstrated that high concentrations of certain metal oxide nanoparticles can disrupt microbial community structure and function, potentially hindering natural biodegradation by indigenous soil bacteria. Similarly, our study suggests that both low and high ZnO-NP concentrations over time can have detrimental effects on bacterial populations, including those crucial for anammox processes. Interestingly, crystalline ZnO-NPs were not detected in the established microcosms after the experiment. However, the mineral hemimorphite (Zn_4_Si_2_O_7_(OH)_2_·2H_2_O) was identified. This suggests potential Zn^2+^ release via dissolution of ZnO-NPs. Indeed, for certain metal-containing nanoparticles (e.g., Ag, ZnO, and CuO), solubilized metal ions are known contributors to their bacterial cytotoxicity [14].

Ag-NPs are widely employed in various industrial, environmental, and medical applications due to their unique properties [14]. However, Ag-NPs are known to exert significant effects on microbial communities [14]. In this study, RT-qPCR analysis revealed a substantial down-regulation (at least 79%) of *hzo* gene expression in sediment samples amended with Ag-NPs of all sizes (50, 100, and 150 nm) at all tested concentrations compared to the unamended control. These findings suggest a severe impact of Ag-NP amendment on the sediment’s microbial community structure, potentially leading to the deterioration of bacterial populations, particularly at high concentrations. Our observations align with Li et al. [58] who reported that high-dose Ag-NPs (10 mg L^-1^) significantly inhibited anammox activity, total nitrogen removal, and the abundance of key enzymes (NIR and HDH) involved in the anammox process. However, these findings diverge from those of Li et al. [58] at low Ag-NP concentrations (1 mg L^-1^), where no significant effects on anammox activity were observed. The observed down-regulation of the *hzo* gene in our study coincides with the potential detrimental effects of Ag-NPs on bacterial populations crucial for petroleum hydrocarbon biodegradation in soil. Additionally, studies have shown that Ag-NP-induced alterations in bacterial community structure can lead to malfunctions in beneficial soil bacteria [14]. Our results contradict those of Z.Z. Zhang et al. [56] who observed no impact on anammox activity with Ag-NP concentrations up to 50 mg g^-1^ suspended solids (SS). Furthermore, they diverge from Meng-wen et al. [59] who reported a positive effect of Ag-NPs on anammox bacteria. In their study, Ag-NPs altered the three-dimensional structure of anammox granules, leading to increased pore size, porosity, and substrate/metal ion diffusion capacity. Additionally, the expression of anammox-related enzymes (NirS, Hdh, and HZS) was upregulated, resulting in enhanced growth rate and nitrogen removal performance of the anammox granules. Notably, crystalline Ag-NPs in microcosms have been shown to undergo oxidation and sulfidation over time, leading to their depletion and susceptibility to environmental transformations [60]. The observed changes in bacterial communities could be a response to this evolving chemical state of Ag. Due to their small size, Ag-NPs exhibit faster kinetics compared to bulk Ag, leading to a significantly reduced lifespan of their metallic state in the environment [61]. Interactions with the soil environment can modify the chemistry of both Ag-NPs and the soil itself, consequently influencing Ag-NP stability, transport, bioavailability, and subsequent toxicity to organisms [61]. Ag-NP dissolution is a crucial environmental behavior, and characterization of Ag^+^ release in soil is essential for understanding the environmental fate, transport, bioavailability, and biological impacts of Ag-NPs [62]. Since Ag-NPs can be oxidized in soil, it remains unclear whether Ag-NPs, Ag^+^ ions, or Ag complexes are the most bioavailable forms [63]. In this study, the observed decrease in anammox activity followed the order Ag-NPs > ZnO-NPs > TiO_2_-NPs, suggesting a correlation between anammox potential and bacterial community structure. The observed inhibition of anammox activity by ZnO and Ag nanoparticles in ammonium-rich water systems is primarily attributed to the release of Zn^2+^ and Ag^+^ ions, respectively. Zn^2+^ has been shown to severely inhibit anammox activity [64], while Ag^+^ can react with organic matter (OM), making it less available for microbial utilization [61]. Both ZnO-NPs and Ag-NPs can be adsorbed by anammox granules, but their impact on anammox activity is primarily due to the release of toxic ions. Zn^2+^ can competitively inhibit ammonium oxidation, while Ag^+^ can enhance OM loading, which has been shown to be detrimental to anammox activity [65]. Additionally, Ag^+^ can increase redox potential (Eh), inhibiting anammox activity by limiting electron donation from ferrous iron and organic compounds [66]. The toxicity of these nanoparticles can also lead to the reduction of extracellular polymeric substances (EPS), compromising the protective capability of anammox granules and increasing the production of reactive oxygen species (ROS), ultimately resulting in decreased anammox activity [67]. The detrimental effects of ZnO-NPs and Ag-NPs on the microbial community likely disrupt the anammox process, as crucial anammox bacteria harboring the *hzo* gene (e.g., *Candidatus Brocadia* and *Candidatus jettenia*) are potentially affected and eliminated from the sediment. This aligns with the established knowledge that most NPs negatively impact natural soil microbial communities [68]. Our findings provide significant evidence that Ag-NPs and ZnO-NPs pose a potential threat to anammox processes in polluted sediments. Conversely, various sediment components might interact with TiO_2_-NPs, potentially reducing their toxicity and even promoting the proliferation of anammox bacteria. The observed inhibition of anammox activity in River Nile sediments by Ag and ZnO nanoparticles aligns with their documented deleterious effects on freshwater and marine ecosystems. Ag-NPs, in particular, can accumulate to concentrations exceeding 0.1 mg L^-1^ in surface waters [69]. Ag-NPs small size and high surface area facilitate rapid ion dissolution, contributing to their toxicity. Moreover, their ability to adsorb biomolecules and interact with cellular receptors can lead to localized toxicity within organisms [70]. Numerous studies have demonstrated the toxicity of Ag-NPs to various aquatic organisms, including algae, invertebrates, and fish. ZnO-NPs have also been shown to be highly toxic to aquatic organisms, with reported toxicity levels as low as 0.2 mg L^-1^ for *Daphnia magna* [71]. The toxicity of ZnO-NPs is primarily attributed to the release of zinc ions (Zn^2+^). While TiO_2_-NPs have been reported to have detrimental effects on freshwater and marine ecosystems, our observed results indicated a positive effect on anammox activity. However, other studies have shown significant toxicity of TiO_2_-NPs to planktonic and biofilm microorganisms, as well as to aquatic invertebrates and vertebrates [72]. The interaction of NPs with co-contaminants can significantly alter their biodistribution, fate, and toxicity profiles. While NPs can form complexes with co-contaminants, leading to synergistic toxicity [73], their effects on aquatic and terrestrial systems are complex and often depend on the specific NP and co-contaminant combination. Analytical techniques for characterizing and quantifying NPs in complex environmental matrices are still under development. The debate on the relevance of metal ion release from NPs for their toxicity continues, while it is evident that NPs can interact with biota through physical pathways, affecting their growth and behavior. NPs have been shown to act as sinks for organic and inorganic co-contaminants, while also influencing the uptake and toxicity of other contaminants [74]. The effects of NP-co-contaminant interactions can vary depending on the specific NP and co-contaminant combination. For example, carbon-based NPs and organic co-contaminants often exhibit antagonistic effects on daphnids and bacteria, while metallic NPs and other chemical stressors can have contradictory effects. TiO_2_-NPs have been shown to both reduce and enhance the uptake of organic and inorganic contaminants, depending on the specific compounds and concentrations. The mechanisms underlying NP-co-contaminant interactions are not fully understood, and further research is needed to assess their relevance in field conditions. While studies have shown that NPs can affect plant growth and microbial communities, the specific effects can vary depending on the NP type, concentration, and plant species. The release of NPs into the environment can have detrimental effects on a wide range of beneficial soil microorganisms. Therefore, well-defined management protocols are necessary to control the use and disposal of nanoparticles to mitigate their adverse effects on microbial communities within various ecosystems.

## Conclusion

This study presents robust evidence for the presence and activity of anammox bacteria within Nile River (Cairo, Egypt) sediments. Amplification and sequencing of 16S rRNA and hydrazine oxidoreductase (*hzo*) genes, coupled with anaerobic enrichment culture experiments, confirmed the occurrence of anammox. Metagenomic analysis of 16S rRNA revealed a diverse anammox community, while PICRUST-based functional prediction identified key anammox biomarkers, hydrazine synthase and hydrazine dehydrogenase. These findings elucidate the role of indigenous microorganisms in sediment-mediated anammox and underscore the potential for enriching specific anammox strains. Importantly, this study demonstrates the predominantly negative impact of Ag, ZnO, and TiO_2_ nanoparticles on anammox activity, as assessed by RT-qPCR analysis of *hzo* gene expression. While most nanoparticle treatments inhibited *hzo* expression, 100 nm TiO_2_ uniquely stimulated anammox activity. These results highlight the potential ecological risks associated with nanoparticle pollution and emphasize the need for comprehensive investigations into nanoparticle-microbe interactions and their consequences for biogeochemical cycling.

## Data Availability

The sequence obtained in this study were deposited into National Center for Biotechnology Information (NCBI) database and are available under accession numbers PP448321 and MW010261.
